# Osteoporosis in Parkinson’s disease and the role of lean body mass: a cross-sectional study in a Brazilian tertiary center

**DOI:** 10.3389/fendo.2024.1326212

**Published:** 2024-04-22

**Authors:** Danielle Pessoa Lima, Francisco Abaeté das Chagas-Neto, João Rafael Gomes de Luna, Yasmin de Oliveira Martins, Samuel Brito de Almeida, Camila Ximenes Feitosa, Leticia Brasil Gradvohl, Isabele Dantas Rosa, Fábia Karine de Moura Lopes, Luciana Felipe Férrer Aragão, Antonio Brazil Viana-Júnior, Kristopherson Lustosa Augusto, Jarbas de Sá Roriz-Filho, Catarina Brasil d’Alva, Renan Magalhães Montenegro-Júnior, Pedro Braga-Neto

**Affiliations:** ^1^ Division of Geriatrics, Department of Clinical Medicine, Universidade Federal do Ceará, Fortaleza, Ceará, Brazil; ^2^ Department of Health Sciences, Medical School of Universidade de Fortaleza, Fortaleza, Ceará, Brazil; ^3^ Clinical Research Unit, Hospital Universitário Walter Cantídio, Universidade Federal do Ceará/EBSERH, Fortaleza, Ceará, Brazil; ^4^ Diagnostic Imaging Department, Division of Radioloy and Sports Medicine of Hospital Geral do Fortaleza, Fortaleza, Ceará, Brazil; ^5^ Diagnostic Imaging Department, TS Health Center, Fortaleza, Ceará, Brazil; ^6^ Division of Endocrinology and Metabolism, Department of Clinical Medicine, Fortaleza, Ceará, Brazil; ^7^ Medical School of Faculty of Universidade Federal do Ceará, Department of Clinical Medicine, Universidade Federal do Ceará (UFC), Fortaleza, Ceará, Brazil; ^8^ Division of Neurology, Clinical Medicine Department, Universidade Federal do Ceará (UFC), Fortaleza, Ceará, Brazil; ^9^ Health Sciences Center, Universidade Federal do Ceará (UFC), Fortaleza, Ceará, Brazil

**Keywords:** osteoporosis, osteopenia, Parkinson’s disease, sarcopenia, falls

## Abstract

**Background:**

Parkinson’s disease (PD) is the second most common neurodegenerative illness and has the highest increase rate in recent years. There is growing evidence to suggest that PD is linked to higher osteoporosis rates and risk of fractures.

**Objective:**

This study aims to estimate the prevalence and factors associated with osteoporosis as defined by the National Osteoporosis Foundation (NOF) and World Health Organization in patients with mild to moderate PD.

**Methods:**

We performed a cross-sectional study at a tertiary public hospital in Fortaleza, Brazil, dating from May 2021 until April 2022. The study sample was comprised of patients with mild to moderate PD who were at least 40 years old and who had the ability to walk and stand unassisted. Bone Mineral Density (BMD) of both the hip (neck of the femur) and the lumbar spine were obtained via properly calibrated Dual Energy X-ray Absorptiometry (DXA) scanning. The FRAX (Fracture Risk Assessment Tool) score was used to determine a person’s 10-year risk of major osteoporotic fracture. The Revised European Working Group on Sarcopenia in Older People (EWGSOP 2) was used as a basis to confirm a sarcopenia diagnosis with the following parameters: low muscle strength gauged by handgrip strength and low muscle quantity by DXA. Physical performance was carefully evaluated by using the Short Physical Performance Battery test. Osteoporosis and osteopenia were diagnosed following the NOF guidelines and WHO recommendations.

**Results:**

We evaluated 107 patients in total, of whom 45 (42%) were women. The group’s mean age was 68 ± 9 years, and the mean disease time span was 9.9 ± 6.0 years and mean motor UPDRS was 43 ± 15. We found that 42.1% and 34.6% of the sample had osteopenia and osteoporosis following NOF criteria, respectively, and 43% and 33.6% following the WHO recommendations. Lower lean appendicular mass was associated to osteopenia and osteoporosis in multinomial logistic regression analysis in both diagnostic criteria.

**Conclusion:**

Our findings provide additional evidence for the protective role of lean mass against osteoporosis in patients with PD.

## Introduction

Parkinson’s disease (PD) is the second most common neurodegenerative illness and has the highest increase rate in recent years (1). It affects 1% of adults over 60 years of age with a rate of 5 and 35 new cases per 100,000 people per year in the world population (2).

PD is clinically characterized by bradykinesia, tremor at rest, rigidity, and progresses to postural instability which predisposes the individual to falls and fractures, resulting in increased hospitalization and institutionalization risk, as well as a lower quality of life (3). Falls are the leading cause of emergency hospital admissions in PD patients and are also a recognized risk factor for fractures (4). In fact, over 90% of all fractures occur following a fall. There is growing evidence to suggest that PD is associated with a higher rate of osteoporosis and fracture risk (5).

Osteoporosis is a metabolic bone disease characterized by decreased bone mass, deterioration of bone microarchitecture, with consequent impairment of bone strength and increased fracture risk (6). According to the World Health Organization (WHO) diagnostic classification, osteoporosis is defined by the history of fractures and/or bone mineral density (BMD) at the femoral neck, total hip or lumbar spine less than or equal to 2.5 standard deviations below the mean BMD of a reference population of young adults measured by dual energy bone densitometry (DXA) (5). However, since the initial publication by the WHO, it has become increasingly evident that many patients are not being diagnosed and do not receive appropriate treatment. Therefore, in order to promote diagnosis and treatment of osteoporosis in postmenopausal women and men aged 50 years or older, the National Osteoporosis Foundation (NOF) proposed the use of the FRAX algorithm (Fracture Risk Assessment Tool) to assess the risk of osteoporosis and fracture aiming to determine the need for BMD testing, spinal imaging and treatment (6) (7).

The prevalence of osteoporosis is 18.3% worldwide. It is silent until the appearance of a fracture, a complication which together with population aging is increasing in prevalence, being expected to reach 160,000 hip fractures per year in Brazil by 2050 (8). Hip fractures are associated with 8 to 36% increase in mortality within 1 year. After a hip fracture episode, 20% of patients require long-term home care and only 40% fully recover their pre-fracture level of independence (8). A large multinational cohort study called “the Global Longitudinal Study of Osteoporosis in Women” (GLOW) was performed to investigate influence of co-morbidities on the risks of fracturing bones, and the strongest association was seen with PD (9).

Given these findings, it appears that osteoporosis is a major public health issue, since it has a very detrimental impact on quality of life and costs. Information regarding the prevalence of osteoporosis in PD patients in Brazil is deficient (10). Furthermore, PD is often neglected by specialists in assessing fracture risk, as they are unaware of the relevance of fractures and osteoporosis in this movement disorder (11). Therefore, the present study aims to assess and discuss the prevalence and risk factors associated with osteoporosis as defined by NOF and WHO in patients with mild to moderate PD in a tertiary center of Northeast Brazil.

## Materials and methods

### Study participants

A cross-sectional study was conducted at a tertiary public hospital in Fortaleza, Brazil, lasting from May 2021 until April 2022. The sample was comprised of patients with PD who had visited the Movement Disorders outpatient clinic of the hospital with varying levels of regularity.

The PD diagnosis was reached by following the Movement Disorders Society (MDS) criteria (12) by a pair of neurologists and one geriatrician specialized in dealing with PD. Participants were required to have a clinical diagnosis of PD and their disease severity score ranging from stage 1 to stage 3 when measured in the modified Hoehn and Yahr (HY) scale (13) to be deemed eligible for the study. Additionally, they were required to be at least 40 years old and have the ability to walk and stand without assistance. Patients with severe disease (HY 4 and 5) were not included due to their limitations, since they would be unfit to complete the Five Times Sit-to-Stand (FTSTS), gait speed or balance tests. Patients with HY 5 are no longer attended to in the outpatient clinic when it comes to face-to-face consultations, since home care is the better option for their situation. Patients who had uncontrolled chronic diseases or severe health conditions that could affect the data interpretation were excluded according to the following criteria: “Heart failure”, specifically the New York Heart Association (NYHA) Functional Classification class III (fatigue caused by less than ordinary activity, dyspnea or palpitation) and IV (symptoms of heart failure even when at rest); Dialysis-dependent renal disease in its end-stage; Neurological diseases (non-PD) that cause motor impairment; Chronic obstructive pulmonary disease at severe (dyspnea at slight exertion) or very severe (dyspnea when at rest and/or during oxygen therapy) levels; Severe wrist, hand, knee or spine osteoarthritis; Cancer diagnosis, except when referring to localized prostate and skin cancer; or Dementia at moderate to severe stages (CDR 2 and 3).

Conditions that would hinder the interpretation of the Dual Energy X-ray Absorptiometry (DXA) were also excluded: Gastrointestinal contrast or radionuclides administered in the last 72 hours; Pregnancy; Heart pacemaker; or Deep Brain Stimulation.

All participants complied in participating by providing written informed consent for the study, which was then sanctioned by the hospital’s Ethics Committee (register number 91075318.1.0000.5045). The researchers conducting the study explained the situation properly to every participant.

### Clinical assessment

An interview was conducted to gather sociodemographic and medical data. The clinical data acquired from the patients were compared with information from their relatives, caregivers, and healthcare records to ensure precision. Data on the antiparkinsonian medications provided by the Brazilian public health system used such as L-dopa, COMT inhibitors (entacapone), MAO-B inhibitors (rasagiline), amantadine, and dopamine agonists (pramipexole), as well as L-dopa formulations such as L-dopa/carbidopa, L-dopa/benserazide, and controlled-release L-dopa formulations were collected. The Schwab and England Activities of Daily Living (SE ADL) Scale was employed to evaluate ADL, the modified Hoehn and Yahr (HY) staging was utilized to assess PD severity, and the Movement Disorders Society-Unified Parkinson’s Disease Rating Scale part III (MDS-UPDRS III) was employed to assess the severity of motor parkinsonian symptoms. Depressive symptoms were evaluated using the 15-item Geriatric Depression Scale (GDS-15), and cognition was assessed through the Mini-Mental Status Examination (MMSE). A fall was defined as “a situation in which the patient inadvertently dropped to the ground or to another lower level, which is not attributable to a seizure, a vehicle or bike accident, or a syncope”. Prior to their medical visit, patients were asked about any similar incidents that occurred in the preceding one and six months earlier. Data on falls gathered from patients were compared with information from relatives, caregivers, and clinical records for accuracy purposes. All participants were weighed while not wearing shoes or holding any heavy accessories such as wallets and mobile phones. The body mass index was calculated by dividing the total body weight (in kilograms) by the square of the height (meters).

### Sarcopenia assessment

The calf is the optimal location for measuring anthropometric parameters to evaluate loss of skeletal muscle mass (SMM) and has been chosen in various studies to estimate SMM (14) (15). Adult lower limbs consist of approximately 30% skeletal muscle. The extremities possess less fat mass than other body regions, thus minimizing their impact on these parameters. The calf perimeter (CP) measurement offers the additional advantage of being feasible, easy to perform, and does not require undressing. The right CP was assessed at the maximum girth of the right calf using a firm but malleable plastic tape while the individual is seated with their knee and ankle at a right angle and feet planted on the floor. The patient disrobed from the lower body to measure the CP on bare skin. There was no compression in the subcutaneous tissues. The right side was selected because Jeong et al. (2020) recently demonstrated that CP measured on the right side exceeded that on the left side, irrespective of hand dominance, in a sample of older adult ambulatory-patients (14). The SARC-F was administered to all patients. The SARC-F is a simple and cost-effective questionnaire employed as an initial screening tool for sarcopenia that is easily implemented in community healthcare settings and other clinical environments. It comprises 5 items to assess the patient’s perception of their physical limitations, including their capacity to walk, lift a 10-pound object, rise from a chair, walk up the stairs and avoid falls (16). The suggestions of the Revised European Working Group on Sarcopenia in Older People (EWGSOP 2) concerning the handgrip strength measure and cut-offs (< 27 kg for men and < 16 kg for women) were adhered to (14). A SAEHAN^®^ dynamometer was employed following the Southhampton protocol, where patients were seated with their forearms resting on the arms of the chair, wrist just over the end of the chair arm, in a neutral position, thumb facing upwards, and their feet flat on the floor. Three attempts on each side were executed, alternating sides, and the maximum grip score from all six attempts was utilized (15). When evaluating physical performance, the Short Physical Performance Battery (SPPB) test was utilized (16). This test consists of evaluations of standing balance, 4-m gait speed, and the time taken to perform five chair stands. During the test, individuals were instructed to maintain their balance by standing with their feet together and spending 10 seconds in each of the semi-tandem and tandem positions. The patient’s gait was measured by having them walk 4 meters at a regular, fast rate. However, the patient had to walk 8 meters, with 2 meters of acceleration and 2 meters of deceleration in addition to the 4 meters. The middle 4 meters were counted, while the first and last 2 meters were ignored (17). In the Five-Times-Sit-to-Stand-Test, patients stood up and sat down as soon as they could while crossing their arms over their chests. The time it took to complete five chair stands was recorded (18). Muscle mass was assessed using DXA to estimate appendicular skeletal muscle mass (ASMM) adjusted for height in meters squared, resulting in the measurement of the lean mass index (LMI = ASMM/Ht2). The lean mass in the arms and legs was used as a basis to gauge appendicular lean mass. For individuals with unilateral harm to body parts, the appendicular lean mass values were acquired by doubling the values for the unaffected side. Low muscle mass was defined as an ASMM index < 7 kg/m^2^ for men and < 5.5 kg/m^2^ for women according to EWGSOP 2 (14).

### Osteoporosis diagnosis

The lumbar spine, femoral neck and total hip bone mineral density (BMD) were measured by a trained radiology technician using a properly calibrated DXA scanner (LUNAR PRODIGY ADVANCE software version 17, model PA+510328, GE Medical Systems). All metallic objects were removed from the participants before starting the scan. The participants were positioned in dorsal decubitus with their body centralized in the scanning area, their palms facing their body and upper and lower limbs extended, and with their arms slightly away from their trunk. A half-body scan called Mirror Image was used for participants who were wider than the scanning table.

The participants remained in dorsal decubitus and centralized in the scanning area, when performing the BMD measurement; however, for the femoral neck measurement, the participants’ legs were turned slightly inwards, and their feet were fixed with Velcro tapes. Then, their legs were elevated with the aid of a foam block for performing the lumbar spine measurement, making a 90° angle. A radiologist of our research group analyzed all the densitometries.

Fragility fractures are defined as those resulting from low energy trauma, i.e. a fall from a high standing position or less, with the skull or face, hands or fingers, feet and toes or patella fractures being excluded (19). The FRAX was created to assess the 10-year absolute probability of hip fracture or major osteoporotic fractures combined (hip, spine, shoulder, or wrist) for an untreated woman or man utilizing widely accessible clinical risk variables for fracture with or without BMD values. FRAX has been tested in the largest number of cohorts (5, 20, 21). The FRAX tool incorporates eight clinical risk factors: previous fragility fracture, parental hip fracture, smoking, systemic glucocorticoid use, excess alcohol consumption, body mass index, rheumatoid arthritis, and other causes of secondary osteoporosis; these, along with age, gender, and BMD of the femoral neck (this input is optional), are used on the 10-year fracture risk estimate (6). The FRAX is a free online tool, and it is the first national fracture-prediction model made for Brazil. It was incorporated into country-wide recommendations for diagnosing and treating postmenopausal osteoporosis in 2017 (7). PD is not included in the FRAX list for calculating the 10-year probability of osteoporotic fractures, and the accuracy of FRAX for these individuals requires additional testing (22). The history of clinical fragility fractures in our study was investigated using a questionnaire that asked about the number and location of prior fractures, as well as their causes and outcomes.

The diagnosis of osteoporosis was performed according to the NOF as follows (6): a hip or vertebral low-trauma fracture (clinically apparent or found on vertebral imaging); and/or T-score ≤−2.5 at the femoral neck, total hip or lumbar spine by DXA; and/or low bone mass (when the T-score is between −1.0 and −2.5 at the total hip, lumbar spine and femoral neck) by DXA with a high probability of a hip fracture or a major fracture related to osteoporosis in the next 10 years (based on the Brazilian model of FRAX; i.e. above the fracture probability of a Brazilian woman with a prior fragility fracture (7). Therefore, according to FRAX Brazil, women with a fracture probability equal to or exceeding that of a woman with previous fragility fracture would be eligible for treatment. Low-trauma or low-energy fractures were considered as diagnostic for osteoporosis (6). Our definition excludes fractures resulting from significant trauma, such as those caused by vehicle accidents or falls from elevated surfaces (23). We also established an osteoporosis diagnosis using the WHO’s criteria: T-score ≤−2.5 at the femoral neck or lumbar spine, and a clinical diagnosis of osteoporosis can be made in the presence of a fragility fracture, notably at the spine, hip, wrist, humerus, rib, and pelvis, without measuring BMD (5).

### Statistical analysis

The study data were collected and managed using the electronic data collection and management tool REDCap hosted at the Clinical Research Unit of the University Hospitals Complex of UFC.

The variables were presented as mean, standard deviation and median, and frequency and prevalence rate. The Mann-Whitney U test and Student’s t tests were used to analyze the participants’ characteristics, verifying non-adherence of the data to the Gaussian distribution. The Pearson’s chi-squared and Fisher’s exact tests were used to investigate the association between categorical variables.

Variables with p < 0.05 entered multinomial analysis in order to find those that were associated independently with osteoporosis, osteopenia and normal bone. We performed Fleiss’ Kappa coefficient to examines the agreement between the NOF and WHO diagnosis. A significance level of 5% was adopted. Statistical analyses were performed using the R statistical program and Microsoft Excel 2016.

## Results

The sample amounted to a total of 107 patients, of which 45 (42%) were women. Their mean age was 68 ± 9 years, mean disease duration was 9.9 ± 6.0 years and mean motor UPDRS was 43 ± 15. For the modified HY, 26 (24.2%) had HY 1 to 2 and 81 (75.7%) had HY 3. According to NOF guidelines, a total of 37 (34.6%) patients had osteoporosis and 45 (42.1%) had osteopenia, while following the WHO recommendations, 36 (33.6%) patients had osteoporosis and 46 (43%) patients had osteopenia.

The mean handgrip strength was 19 ± 6 kg in women and 34 ± 9 kg in men. In addition, 51 (48%) patients had positive screening for sarcopenia according to the SARC-F and the prevalence of confirmed sarcopenia was 11 (N=10.3%). The most prevalent comorbidities were hypertension (N=51, 47.6%) and type 2 diabetes mellitus (n=14, 13.1%). When we inquired about falls during the last 6 months of the consultation, 47 (39.8%) patients reported having fallen at least once, while 31 (26.3%) had experienced two or more falls. Moreover, 14 (13.0%) patients with PD had 22 prior fragility fractures and, fractures were multiple in five (4.67%) of them. Fracture bone sites were wrist (n=8; 36.36%), costs (n=5; 22.72%), clavicle (n=4; 18.18%), shoulder (n=3; 13.63%), pelvis (n=1; 4.54%) and elbow (n=1; 4.54%).

**Table 1** indicates the clinical features and the main demographic of those who participated in the study, and also the univariate analysis for osteoporosis and osteopenia according to the NOF guidelines. The following variables were associated with osteoporosis based on the NOF guidelines: female gender, lower handgrip strength, lower body mass index, lower appendicular skeletal muscle mass index, lower calf circumference, and lower gait speed. These variables entered the multinomial logistic regression model (**Table 2**) and only lower appendicular skeletal muscle mass index was independently associated with osteoporosis with statistical significance. The multinomial logistic regression model did not include calf circumference since it has a high correlation with another variable in the analysis, appendicular lean mass.

**Table 1:** Sociodemographic and clinical traits and bivariate analysis of osteoporosis and osteopenia according to NOF.

		NOF			p
Variables	N	Osteoporosis N = 37^1^	Osteopenia N = 45^1^	Normal N = 25^1^	
**Gender**	107				**<0.001**
Female		23 (62%)	19 (42%)	3 (12%)	
Male		14 (38%)	26 (58%)	22 (88%)	
**Age**	107	69 ± 9 (70)	68 ± 10 (69)	65 ± 8 (63)	0.172
**Hypertension**	107	14 (38%)	24 (53%)	13 (52%)	0.333
**Diabetes**	107	4 (11%)	6 (13%)	4 (16%)	0.876
**Depression**	107	6 (16%)	6 (13%)	2 (8.0%)	0.662
**Schwab-England**	107	84 ± 14 (90)	85 ± 9 (90)	86 ± 10 (90)	0.986
**SARC-F score**	106	4.70 ± 2.9 (5.00)	3.25 ± 2.47 (3.00)	3.92 ± 3.04 (3.00)	0.089
**Handgrip strength**	104	23 ± 9 (20)	28 ± 10 (27)	34 ± 10 (32)	<0.001
**Total SPPB**	104	8.19 ± 3.02 (9.00)	9.14 ± 2.60 (9.00)	9.36 ± 2.02 (10.00)	0.128
**BMI**	107	24.9 ± 4.3 (23.5)	25.7 ± 4.0 (26.3)	28.4 ± 4.6 (27.4)	**0.020**
**ALM/HT^2^**	106	6.60 ± 1.12 (6.57)	7.16 ± 1.01 (6.91)	8.35 ± 1.15 (8.33)	**<0.001**
**Calf circumference**	104	32.1 ± 4.1 (32.0)	33.5 ± 2.7 (33.0)	34.9 ± 3.8 (34.5)	**0.044**
**Calcium Intake**	90	945 ± 1,570 (633)	714 ± 300 (719)	686 ± 253 (689)	0.685
**UPDRS Part 3**	107	45 ± 14 (45)	42 ± 15 (41)	44 ± 18 (37)	0.695
**GDS**	105	5.14 ± 3.37 (4.00)	4.73 ± 3.32 (4.00)	4.58 ± 3.61 (4.00)	0.772
**MEEM**	103	23.0 ± 4.8 (24.0)	24.4 ± 3.8 (26.0)	24.6 ± 4.0 (25.0)	0.262
**Disease Duration**	107	10.7 ± 6.8 (10.0)	9.6 ± 5.7 (10.0)	9.4 ± 5.2 (7.0)	0.774
**Sarcopenia**	104	6 (16%)	4 (8.9%)	1 (4.2%)	0.365
**Hoehn Yahr**	107				0.634
Mild		8 (22%)	13 (29%)	5 (20%)	
Moderate		29 (78%)	32 (71%)	20 (80%)	
**Smoking**	107	2 (5.4%)	0 (0%)	0 (0%)	0.170
**Alcoholism**	107	5 (14%)	7 (16%)	2 (8.0%)	0.711
**Gait speed**	102	1.16 ± 0.47 (1.21)	1.50 ± 0.58 (1.40)	1.41 ± 0.40 (1.48)	0.036
**PIGD**	107	9.70 ± 2.49 (10.00)	9.27 ± 2.77 (10.00)	8.64 ± 2.41 (9.00)	0.311

^1^ n (%); mean ± standard deviation (median); ^2^ Chi-squared test; Kruskal-Wallis test; Fisher’s exact test; SPPB, Short Physical Performance Battery; BMI, Body Mass Index; ALM/HT^2^, appendicular skeletal muscle mass (ASMM) which was adjusted for height in meters squared; MDS-UPDRS Part III score, Movement Disorders Society-Unified Parkinson’s Disease Rating Scale part III; GDS-15, Geriatric Depression Scale; MMSE, Mini-Mental State Examination; PIGD, Postural instability and gait dysfunction subtype. Bold value indicates p<0.05.

**Table 2:** Multinomial logistic regression of diagnosis of osteoporosis and osteopenia according to NOF.

Outcome	Variables	OR^1^	95%CI^1^	P-value
**Osteoporosis**	Gender			
	Female	—	—	
	Male	1.50	0.28, 7.90	0.635
	Handgrip strength	0.98	0.89, 1.08	0.684
	ALM_HT2	0.25	0.11, 0.60	**0.002**
	Gait speed	0.39	0.09, 1.65	0.200
**Osteopenia**	Gender			
	Female	—	—	
	Male	1.22	0.27, 5.46	0.798
	Handgrip strength	0.99	0.91, 1.07	0.720
	ALM_HT2	0.43	0.21, 0.88	**0.021**
	Gait speed	1.16	0.33, 4.07	0.816

^1^OR, Odds Ratio; CI, Confidence Interval.

ALM/HT^2^, appendicular skeletal muscle mass (ASMM) that was adjusted for height in meters squared. Bold value indicates p<0.05.

**Table 3** shows the univariate analysis for osteoporosis and osteopenia according to the WHO recommendations. The following variables were associated with osteoporosis based on WHO recommendations: female gender, lower handgrip strength, lower body mass index, lower appendicular skeletal muscle mass index, lower calf circumference and lower gait speed. These variables entered the multinomial logistic regression model (**Table 4**) and only lower appendicular skeletal muscle mass index was independently associated with osteoporosis with statistical significance. The multinomial logistic regression model did not include calf circumference since it has a high correlation with another variable in the analysis, appendicular lean mass.

**Table 3:** Sociodemographic and clinical traits and bivariate analysis for osteoporosis and osteopenia according to the WHO.

		Osteoporosis WHO			p^2^
Variables	N	Osteoporosis N = 36^1^	Osteopenia N = 46^1^	Normal N = 25^1^	
**Gender**	107				**<0.001**
Female		26 (72%)	17 (37%)	2 (8.0%)	
Male		10 (28%)	29 (63%)	23 (92%)	
**Age**	107	68 ± 9 (70)	68 ± 10 (72)	65 ± 9 (63)	0.269
**Hypertension**	107	15 (42%)	23 (50%)	13 (52%)	0.668
**Diabetes**	107	5 (14%)	4 (8.7%)	5 (20%)	0.379
**Depression**	107	7 (19%)	5 (11%)	2 (8.0%)	0.407
**Schwab-England**	107	83 ± 14 (90)	85 ± 10 (90)	86 ± 10 (90)	0.880
**SARC-F score**	106	4.39 ± 2.93 (4.00)	3.42 ± 2.53 (3.00)	4.12 ± 3.07 (3.00)	0.357
**Handgrip strength**	104	22 ± 9 (18)	28 ± 10 (28)	35 ± 10 (32)	**<0.001**
**Total SPPB**	104	8.19 ± 2.89 (9.00)	9.16 ± 2.73 (9.00)	9.28 ± 2.03 (10.00)	0.335
**BMI**	107	24.9 ± 4.1 (24.8)	25.4 ± 4.0 (25.7)	28.9 ± 4.5 (27.7)	**0.004**
**ALM/HT^2^**	106	6.44 ± 1.06 (6.35)	7.20 ± 0.99 (6.98)	8.50 ± 0.95 (8.42)	**<0.001**
**Calf circumference**	104	31.8 ± 3.8 (31.3)	33.5 ± 3.1 (33.5)	35.3 ± 3.5 (35.0)	**0.003**
**Calcium Intake**	90	723 ± 409 (648)	895 ± 144 (683)	706 ± 264 (699)	0.925
**MDS- UPDRS Part 3**	107	44 ± 15 (45)	43 ± 14 (42)	43 ± 18 (37)	0.901
**GDS**	105	4.69 ± 2.95 (4.00)	5.00 ± 3.61 (4.00)	4.75 ± 3.63 (4.50)	0.983
**MEEM**	103	23.6 ± 3.8 (24.0)	23.8 ± 4.7 (26.0)	24.7 ± 4.0 (26.0)	0.404
**Disease duration**	107	10.6 ± 6.8 (9.5)	9.7 ± 5.8 (10.0)	9.4 ± 5.2 (7.0)	0.840
**Sarcopenia**	106	6 (17%)	5 (11%)	0 (0%)	0.101
**Hoehn Yahr**	107				0.843
Mild		9 (25%)	12 (26%)	5 (20%)	
Moderate		27 (75%)	34 (74%)	20 (80%)	
**Smoking**	107	1 (2.8%)	1 (2.2%)	0 (0%)	>0.999
**Alcoholism**	107	5 (14%)	7 (15%)	2 (8.0%)	0.712
**Gait speed**	102	1.15 ± 0.46 (1.17)	1.50 ± 0.58 (1.43)	1.40 ± 0.41 (1.48)	**0.019**
**PIGD**	107	9.56 ± 2.53 (9.50)	9.35 ± 2.76 (10.00)	8.72 ± 2.42 (9.00)	0.492

^1^ n (%); mean ± standard deviation (median); ^2^ Chi-squared test; Kruskal-Wallis test; Fisher’s exact test; SPPB, Short Physical Performance Battery; BMI, Body Mass Index; ALM/HT^2^, appendicular skeletal muscle mass (ASMM) which was adjusted for height in meters squared; MDS-UPDRS Part III score, Movement Disorders Society-Unified Parkinson’s Disease Rating Scale part III; GDS-15, Geriatric Depression Scale; MMSE, Mini-Mental State Examination; PIGD, Postural instability and gait dysfunction subtype. Bold value indicates p<0.05.

**Table 4:** Multinomial logistic regression of diagnosis of osteoporosis and osteopenia according to WHO.

Outcome	Variables	OR^1^	95%CI^1^	P-value
**Osteoporosis**	Gender			
	Female	—	—	
	Male	0.78	0.13, 4.54	0.784
	Handgrip strength	1.01	0.92, 1.12	0.772
	ALM_HT2	0.14	0.05, 0.40	**<0.001**
	Gait speed	0.35	0.07, 1.69	0.191
**Osteopenia**	Gender			
	Female	—	—	
	Male	1.49	0.31, 7.21	0.617
	Handgrip strength	0.99	0.91, 1.07	0.798
	ALM_HT2	0.33	0.15, 0.72	**0.005**
	Gait speed	1.04	0.28, 3.84	0.950

^1^OR, Odds Ratio; CI, Confidence Interval; ALM/HT^2^, appendicular skeletal muscle mass (ASMM) that was adjusted for height in meters squared. Bold value indicates p<0.05.

**Table 5** presents overall agreement results between the NOF and WHO diagnosis by Fleiss’ kappa coefficient. There was substantial overall agreement between both diagnostic criteria for normal, osteopenia and osteoporosis in this sample. **Table 6** shows patients who received different diagnoses based on the NOF and WHO diagnostic criteria.

**Table 5:** Fleiss’s kappa coefficient test for different diagnostic criteria.

Diagnosis	Kappa	p-value
Global agreement	0.853	<0.001
Normal	0.954	<0.001
Osteopenia	0.84	<0.001
Osteoporosis	0.779	<0.001

NOF, National Osteoporosis Foundation; WHO, World Health Organization; BMD, Bone mineral densitometry.

**Table 6:** Differences between diagnosis according to NOF and WHO criteria.

PATIENT IDENTIFICATION	FRAX	NOF CRITERIA	WHO CRITERIA
**7**	NO TREATMENT	OSTEOPENIA	OSTEOPOROSIS
**18**	OSTEOPOROSIS	OSTEOPOROSIS	OSTEOPENIA
**27**	NO TREATMENT	OSTEOPENIA	OSTEOPOROSIS
**72**	OSTEOPOROSIS	OSTEOPOROSIS	NORMAL
**99**	NO TREATMENT	OSTEOPENIA	OSTEOPOROSIS
**111**	OSTEOPOROSIS	OSTEOPOROSIS	OSTEOPENIA
**131**	NO TREATMENT	NORMAL	OSTEOPOROSIS
**132**	OSTEOPOROSIS	OSTEOPOROSIS	OSTEOPENIA
**133**	NO TREATMENT	OSTEOPENIA	OSTEOPOROSIS
**137**	OSTEOPOROSIS	OSTEOPOROSIS	OSTEOPENIA
**145**	OSTEOPOROSIS	OSTEOPOROSIS	OSTEOPENIA

**Figure 1:**
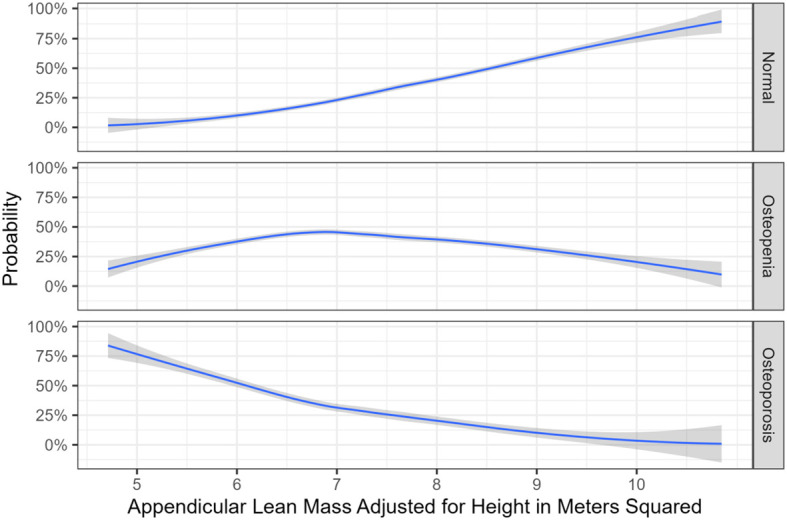
Association between appendicular lean mass which was adjusted for height in meters squared, as well as bone mass.

**Figure 1** illustrates the association of osteoporosis, osteopenia and normal bone mass according to the NOF guidelines with appendicular lean mass adjusted for height in meters squared. **Table 7** displays the fracture locations for each patient who positively answered to a prior fracture in the FRAX questionnaire.

**Table 7:** Fracture sites.

Patients	Fracture sites
FFS	Shoulder and knee
FCF	Forearm 3 times
LCA	Clavicle
RAN	Wrist
FGF	Pelvis
FRM	Wrist and shoulder
MLSG	Elbow
RRV	Forearm
ASS	Forearm and clavicle
MAV	Five ribs at different times
MNGP	Shoulder
JOS	Clavicle
FVFS	Clavicle
FCRF	Wrist

According to **Tables 1** and **3**, patients with PD and osteoporosis exhibit lower BMI, reduced handgrip strength, decreased appendicular muscle mass (resulting in smaller calf circumference), and slower gait speed. These factors together point to indicators of frailty which is very prevalent in PD patients (24).

## Discussion

The goal of the present study was to assess the pervasiveness of osteopenia and osteoporosis in PD patients who are dealing with HY stages 1 to 3, which were found to be 42.1% and 34.6% following NOF criteria, respectively, and were found to be 43% and 33.6% following the WHO recommendations.

The prevalence of osteoporosis and osteopenia was quite similar between the NOF and WHO criteria in our study. While the NOF considers a more comprehensive evaluation of fracture risk using the FRAX instrument (6), the WHO is primarily concerned with BMD and the presence of osteoporotic fractures (5). The choice between the two sets of criteria is based on the doctor’s preferences and the clinical information provided by the patient. Six individuals in our sample had FRAX values indicating a high 10-year risk of fracture but no history of fractures, as well as osteopenia by DXA. Thus, the WHO labeled these patients as having osteopenia, but the NOF diagnosed them with osteoporosis. Meanwhile, four individuals with densitometric osteopenia suffered minor low-impact fractures, but had a low 10-year fracture risk based on FRAX score, with no need for treatment.

Overall, many factors may play a role in the development of bone loss associated PD, such as reduced mobility, malnutrition, low BMI, decreased muscular strength, drug usage, as well as insufficiency of vitamin D (25). Torsney et al. (26) demonstrated a higher association between PD with osteoporosis and osteopenia compared to healthy adults. The OR combination for osteoporosis in PD was 2.61 (1.69-4.03).

Ozturk et al. (25) managed to show that the lumbar and femoral BMD levels were inferior in the advanced stages and also in the early stages of PD, including HY 1–1.5. It is logical to deduce that bone mass might be impacted prior to the manifestation of motor symptoms, specifically during the prodromal period of PD. Furthermore, it can be proposed that PD is not only a chronic neurodegenerative disorder, but also a systemic catabolic process that impacts the musculoskeletal system (25). It is also suggested that trunk muscular strength is intimately linked to lumbar spine BMD, and there is an independent association between leg muscle strength and hip BMD in female patients (26). Additionally, alongside reduced axial mobility, postural changes such as anteflexion caused by increased abdominal rigidity with disease progression not only provoke festination, but also diminish axial loading on the lumbar spine due to forward displacement of the center of gravity. By reducing mechanical pressure on vertebrae, the decline in axial loading on lumbar vertebrae in an erect posture may potentially contribute to a low lumbar spine BMD (27).

Considering the patients with osteoporosis and osteopenia from our sample, 60% and 43% were woman, respectively. Most patients in our sample were male, aligning with studies that indicate a higher prevalence of the disease in males (27). The main reasons are that men tend to exhibit greater bone density. Females also experience bone loss at a younger age and a more accelerated pace than males, resulting in higher levels of bone resorption markers. These factors collectively lead to women who are 50 or older showing a fourfold higher osteoporosis rate and a twofold higher osteopenia rate compared to men (28).

Gao et al. (29) discovered that BMD in PD patients was significantly lower when compared to healthy controls during a cross-sectional study of 54 patients dealing PD and 59 age-matched controls. According to this study, the BMD scores of the total hip, lumbar and the femoral neck were reduced in females compared to males in the healthy group. Females in the PD group exhibited notably decreased BMD in the hip compared to males. They also identified a significant negative correlation between BMD in the femoral neck, lumbar spine and total hip and the overall severity of PD. In our study, 78% of osteoporosis patients had moderate-stage disease (HY 2.5-3), while 22% had mild-stage disease (HY 1-2).

Lorefalt et al. (30) discovered that BMD in the entire body, femoral neck and total hip was lower and declined throughout the examined year in 26 PD patients compared to 26 age and sex-matched healthy controls. Reduced body weight and decreased physical activity were identified as risk factors for low BMD in PD, while stiffness appeared to be rather protective. Schneider et al. (31) researched a cohort study that examined 8,105 older women with known PD status (n = 73 with PD) for six years. In comparison to women without PD, age-adjusted mean total hip BMD scored 7.3% lower in women with PD. Evatt et al. (32) determined that vitamin D deficiency was significantly more prevalent in PD patients (55%) than in age-matched healthy controls (36%) and those with Alzheimer’s disease (41%). It is important to note that PD patients have inadequate calcium intake and minimal sun exposure (32).

A cross-sectional observational study conducted on subjects ≥20 years based on the Korea National Health and Nutrition Examination Survey (KNHANES) (33) demonstrated that physical and anthropometric composition parameters showed remarkable correlation with BMD in all age groups for both men and women. Data from the KNHANES (2005–2008), answered by adults in their 50s and older (n = 3.296), indicated that each increment in BMI was linked to a rise of 0.0082 g/cm^2^ in BMD, and a 10-unit increment in BMI can aid in restoring BMD levels back to normal.

Saarelainen et al. (34) discovered that women with a BMI of 20 kg/m^2^ had diminished bone mass at the femoral neck and the spine at 2 and 4 years after their menopause, respectively, while women with a BMI of 30 kg/m^2^ showed diminished bone mass at the femoral neck and the spine at 5 and 9 years after their menopause, respectively.

Our study also found a statically significant association between lower lean appendicular mass and osteoporosis in multinomial logistic regression analysis. This finding is an important contribution to the growing body of research as it suggests the critical role of muscle quality in bone health (35).

The mechanism underlying this association could be related to the mechanical loading exerted by muscles on bones, thereby stimulating bone formation and decreasing the risk of osteoporosis (28). Furthermore, muscles and bones share common anabolic pathways, and muscle-derived factors, such as irisin, may directly influence bone metabolism. The protective role of lean mass could therefore be attributed to these mechanical and biochemical interactions between muscles and bones (36).

This study also sheds light on the specific population of PD patients who are known to be at higher risk for osteoporosis due to factors like physical inactivity, vitamin D deficiency, and direct neurodegenerative effects on bone metabolism (37). While these findings are promising, it is important to consider that the handgrip strength test is a surrogate measure of overall muscle mass and not a direct measure. Although it has been widely used and validated in the research setting, it may not fully represent the complex interplay of factors that contribute to bone health, such as regional differences in muscle and bone mass (38–41).

The association we found among osteoporosis and calf circumference, gait speed and handgrip strength are well-established parameters for assessing muscle and bone health (42). As muscle wasting is a well-recognized problem in PD (43), interventions that can help maintain or increase muscle mass may not only improve physical functioning, but also protect against osteoporosis. This underscores the undeniable need of comprehensive care for patients who struggle with PD, which should not only include pharmacological therapy but also physical and nutritional interventions (44).

It is worth noting that some studies have focused on identifying predictive factors for imminent fractures, meaning fractures expected to occur within the next 2 years, and found that poorer health status, slower walking speed, the presence of comorbidities, and a higher risk of falls are all predictors of an imminent fracture risk (45–47). An imminent fracture risk corresponds to a very high risk of fracture, warranting consideration of anabolic therapy. We argue whether advanced PD, being a condition associated with frailty and high risk of falls, should not be considered as a predictor of imminent risk for fracture (48–50). In essence, the association of these findings (such as appendicular lean mass and gait speed) with osteoporosis within PD patients suggests a potential need for more effective osteoporosis treatments, specifically anabolic therapies (20).

Furthermore, according to numerous authors and the consensus of the American College of Clinical Endocrinologists (AACE), the risk of falls is another critical aspect that categorizes patients as having a very high risk (51, 52). The incidence of falls experienced by these patients in the past year was nearly 40%, underscoring the critical need for fracture prevention in this population.

In summary, we found that approximately 33.6-34.6% of these patients have osteoporosis, with 14 patients (13%) having a history of fractures alongside sarcopenia, reduced gait speed, and an increased propensity to falls.

Nevertheless, our study does have some limitations. Firstly, the cross-sectional nature of the present study reduces its ability to establish causal relationships. Longitudinal studies are necessary to confirm the protective effect of lean mass on bone health in patients with PD. Secondly, as mentioned earlier, handgrip strength is a surrogate measure of overall muscle strength. Future studies should consider using more direct measures of muscle quality. Thirdly, severe disease cases (HY 4-5) were not included in the study, therefore our findings refer to mild to moderate disease (HY 1-3). Fourthly, we did not perform a vertebral fracture assessment (VFA) or conventional lateral thoracic and lumbar spine X-ray to investigate subclinical morphometric vertebral fractures.

Despite these limitations, our findings contribute to a growing body of evidence suggesting that lean mass may play a critical role in bone health, particularly in populations which are at risk for osteoporosis, like those with PD. They highlight the need for further research in this area, both to confirm these findings and to identify effective interventions for improving muscle mass and bone health in these patients.

In conclusion, our findings provide additional evidence that lean mass may have a protective role against osteoporosis in patients with PD. Future research should continue to explore the implications of these findings on preventing and managing osteoporosis in this population, with a focus on interventions that can enhance muscle mass and strength.

## Data availability statement

The raw data supporting the conclusions of this article will be made available by the authors, without undue reservation.

## Ethics statement

The studies involving humans were approved by Hospital Universitário Walter Cantidio. The studies were conducted in accordance with the local legislation and institutional requirements. The participants provided their written informed consent to participate in this study.

## Author contributions

DL: Writing – original draft, Supervision, Project administration, Methodology, Investigation, Data curation, Conceptualization. FC-N: Writing – original draft, Investigation. JG: Writing – original draft, Investigation. YM: Writing – original draft, Investigation. SA: Writing – original draft, Conceptualization. CF: Writing – original draft, Investigation. LG: Writing – original draft, Investigation. IR: Writing – original draft, Investigation. FL: Writing – original draft, Investigation. LA: Writing – original draft, Investigation. AV-J: Writing – original draft, Formal Analysis. KA: Writing – original draft, Conceptualization. JR-F: Writing – original draft, Supervision. CD’A: Writing – original draft, Supervision. RM-J: Writing – original draft, Supervision, Funding acquisition. PB-N: Writing – original draft, Supervision, Funding acquisition.

## References

[1] Bloem BR Okun MS Klein C. Parkinson’s disease. Lancet. (2021) 397:2284–303. doi: 10.1016/S0140-6736(21)00218-X 33848468

[2] Brundin P. Parkinson disease epidemiology, pathology, genetics and pathophysiology. Physiol Behav. (2017) 176:139–48. doi: 10.1016/j.cger.2019.08.002.Parkinson PMC6905381 31733690

[3] Lamont RM Morris ME Menz HB McGinley JL Brauer SG. Falls in people with Parkinson’s disease: A prospective comparison of community and home-based falls. Gait Posture. (2017) 55:62–7. doi: 10.1016/j.gaitpost.2017.04.005 28419875

[4] Raglione LM Sorbi S Nacmias B. Osteoporosis and parkinson’s disease. Clin cases Miner Bone Metab. (2011) 8:16–8. doi: 10.1155/2012/127362 PMC3279061 22461823

[5] Kanis JA. Assessment of osteoporosis at the primary health care level Vol. 339. UK: World Health Organization Collaborating Centre for Metabolic Bone Diseas, UK (2007). Available at: www.shef.ac.uk/FRAX/pdfs/WHO_Technical_Report.pdf.

[6] Cosman F de Beur SJ LeBoff MS Lewiecki EM Tanner B Randall S. Clinician’s guide to prevention and treatment of osteoporosis. Osteoporos Int. (2014) 25:2359–81. doi: 10.1007/s00198-014-2794-2 PMC4176573 25182228

[7] Zerbini CAF Szejnfeld VL Abergaria BH McCloskey EV Johansson H Kanis JA. Incidence of hip fracture in Brazil and the development of a FRAX model. Osteoporos Int. (2017) 28:1765–1769. doi: 10.1007/s11657-015-0224-5 26303038

[8] Salari N Ghasemi H Mohammadi L hasan BM Rabieenia E Shohaimi S. The global prevalence of osteoporosis in the world: a comprehensive systematic review and meta-analysis. J Orthop Surg Res. (2021) 16. doi: 10.1186/s13018-021-02772-0 PMC8522202 34657598

[9] Dennison EM Compston JE Flahive J Siris ES Gehlbach SH Adachi JD. Effect of co-morbidities on fracture risk: Findings from the Global Longitudinal Study of Osteoporosis in Women (GLOW). Bone. (2012) 50:1288–93. doi: 10.1016/j.bone.2012.02.639 PMC4886309 22426498

[10] Aziziyeh R Amin M Habib M Perlaza JG McTavish RK Lüdke A. A scorecard for osteoporosis in four Latin American countries: Brazil, Mexico, Colombia, and Argentina. Arch Osteoporos. (2019) 14:1–10. doi: 10.1007/s11657-019-0622-1 31250192

[11] Lyell V Henderson E Devine M Gregson C. Assessment and management of fracture risk in patients with Parkinson’s disease. Age Ageing. (2015) 44:34–41. doi: 10.1093/AGEINGAFU122 25236847

[12] Postuma RB Berg D Stern M Poewe W Olanow CW Oertel W. MDS clinical diagnostic criteria for Parkinson’s disease. Mov Disord. (2015) 30(12):1591–601. doi: 10.1002/mds.26424 26474316

[13] Goetz CG Poewe W Rascol O Sampaio C Stebbins GT Counsell C. Movement Disorder Society Task Force report on the Hoehn and Yahr staging scale: Status and recommendations. Mov Disord. (2004) 19:1020–8. doi: 10.1002/mds.20213 15372591

[14] Cruz-Jentoft AJ Bahat G Bauer J Boirie Y Bruyère O Cederholm T. Sarcopenia: Revised European consensus on definition and diagnosis. Age Ageing. (2019) 48:16–31. doi: 10.1093/ageing/afy169 30312372 PMC6322506

[15] Vetrano DL Pisciotta MS Laudisio A Lo Monaco MR Onder G Brandi V. Sarcopenia in parkinson disease: comparison of different criteria and association with disease severity. J Am Med Dir Assoc. (2018) 19:523–7. doi: 10.1016/j.jamda.2017.12.005 29396191

[16] de Fátima Ribeiro Silva C Ohara DG Matos AP Pinto ACPN Pegorari MS. Short physical performance battery as a measure of physical performance and mortality predictor in older adults: A comprehensive literature review. Int J Environ Res Public Health. (2021) 18. doi: 10.3390/ijerph182010612 PMC8535355 34682359

[17] Combs SA Diehl MD Filip J Long E. Short-distance walking speed tests in people with Parkinson disease: Reliability, responsiveness, and validity. Gait Posture. (2014) 39:784–8. doi: 10.1016/j.gaitpost.2013.10.019 24246801

[18] Duncan RP Leddy AL Earhart GM. Five times sit-to-stand test performance in Parkinson’s disease. Arch Phys Med Rehabil. (2011) 92:1431–6. doi: 10.1016/j.apmr.2011.04.008 PMC3250986 21878213

[19] NICE Guideline. Osteoporosis: assessing the risk of fragile fractures. Available at: www.nice.org.uk/guidance/cg146(2012) [Accessed October 15, 2023].

[20] Compston JE Drake MT. Defining very high fracture risk: is FRAX fit for purpose? J Bone Miner Res. (2020) 35:1399–403. doi: 10.1002/jbmr.4134 32696997

[21] Compston JE McClung MR Leslie WD. Osteoporosis seminar 2019. Lancet. (2019) 393:364–76. doi: 10.1016/S0140-6736(18)32112-3 30696576

[22] Schini M Bhatia P Shreef H Johansson H Harvey NC Lorentzon M. Increased fracture risk in Parkinson’s disease - An exploration of mechanisms and consequences for fracture prediction with FRAX. Bone. (2023) 168. doi: 10.1016/j.bone.2022.116651 36574893

[23] LeBoff MS Greenspan SL Insogna KL Lewiecki EM Saag KG Singer AJ. The clinician’s guide to prevention and treatment of osteoporosis. Osteoporos Int. (2022) 33:2049–102. doi: 10.1007/s00198-021-05900-y PMC9546973 35478046

[24] Zheng Z Lv Y Rong S Sun T Chen L. Physical frailty, genetic predisposition, and incident parkinson disease. JAMA Neurol. (2023) 80:455–61. doi: 10.1001/jamaneurol.2023.0183 PMC10012040 36912851

[25] Malochet-Guinamand S Franck Durif TT. Parkinson’s disease: A risk factor for osteoporosis. Jt Bone Spine. (2015) 82:406–10. doi: 10.1016/j.jbspin.2015.03.009 26453100

[26] Torsney KM Noyce AJ Doherty KM Bestwick JP Dobson R Lees AJ. Bone health in Parkinson ’ s disease : a systematic review and meta-analysis. J Neurol Neurosurg Psychiatry. (2014) 85:1159–66. doi: 10.1136/jnnp-2013-307307 PMC4173751 24620034

[27] Cerri S Mus L Blandini F. Parkinson’s disease in women and men: what’s the difference? J Parkinsons Dis. (2019) 9:501–15. doi: 10.3233/JPD-191683 PMC6700650 31282427

[28] Frost HM. On our age-related bone loss : insights from a new paradigm. J Bone Miner Res. (1997) 12:9–13. doi: 10.1359/jbmr.1997.12.10.1539 9333113

[29] Gao H Wei X Liao J Wang R Xu J Liu X. Lower bone mineral density in patients with Parkinson’s disease: A cross-sectional study from Chinese Mainland. Front Aging Neurosci. (2015) 7:1–9. doi: 10.3389/fnagi.2015.00203 26578949 PMC4621433

[30] Lorefält B Toss G Granérus AK. Bone mass in elderly patients with Parkinson’s disease. Acta Neurol Scand. (2007) 116:248–54. doi: 10.1111/j.1600-0404.2007.00875.x 17824904

[31] Schneider JL Fink HA Ewing SK Ensrud KE Cummings SR. The association of Parkinson’s disease with bone mineral density and fracture in older women. Osteoporos Int. (2008) 19:1093–7. doi: 10.1007/s00198-008-0583-5 18301855

[32] Evatt ML DeLong MR Khazai N Rosen A Triche S Tangpricha V. Prevalence of vitamin D insufficiency in patients with Parkinson disease and Alzheimer disease. Arch Neurol. (2008) 65:1348–52. doi: 10.1001/archneur.65.10.1348 PMC2746037 18852350

[33] Kim YM Kim SH Kim S Yoo JS Choe EY Won YJ. Variations in fat mass contribution to bone mineral density by gender, age, and body mass index: The Korea National Health and Nutrition Examination Survey (KNHANES) 2008–2011. Osteoporos Int. (2016) 27:2543–54. doi: 10.1007/s00198-016-3566-y 27112764

[34] Saarelainen J Kiviniemi V Kröger H Tuppurainen M Niskanen L Jurvelin J. Body mass index and bone loss among postmenopausal women: The 10-year follow-up of the OSTPRE cohort. J Bone Miner Metab. (2012) 30:208–16. doi: 10.1007/s00774-011-0305-5 21938384

[35] Binkley N Buehring B. Beyond FRAX®: it’s time to consider “Sarco-osteopenia. J Clin Densitom. (2009) 12:413–6. doi: 10.1016/j.jocd.2009.06.004 19733110

[36] Colaianni G Sanesi L Storlino G Brunetti G Colucci S Grano M. Irisin and bone : from preclinical studies to the evaluation of its circulating levels in different populations of human subjects. Cells. (2019) 15:451. doi: 10.3390/cells8050451 PMC6562988 31091695

[37] Bezza A Ouzzif Z Naji H Achemlal L Mounach A Nouijai M. Prevalence and risk factors of osteoporosis in patients with Parkinson’s disease. Rheumatol Int. (2008) 28:1205–9. doi: 10.1007/s00296-008-0632-6 18592245

[38] Roberts HC Denison HJ Martin HJ Patel HP Syddall H Cooper C. A review of the measurement of grip strength in clinical and epidemiological studies: Towards a standardised approach. Age Ageing. (2011) 40:423–9. doi: 10.1093/ageing/afr051 21624928

[39] Yao R Yao L Yuan C Gao BL. Accuracy of calf circumference measurement, SARC-F questionnaire, and ishii’s score for screening stroke-related sarcopenia. Front Neurol. (2022) 13:1–10. doi: 10.3389/fneur.2022.880907 PMC9099210 35572926

[40] Singh R Gupta S. Relationship of calf circumference with bone mineral density and hip geometry: a hospital-based cross-sectional study. Arch Osteoporos. (2015) 10. doi: 10.1007/s11657-015-0221-8 26085340

[41] Lin YH Chen HC Hsu NW Chou P Teng MMH. Hand grip strength in predicting the risk of osteoporosis in Asian adults. J Bone Miner Metab. (2021) 39:289–94. doi: 10.1007/s00774-020-01150-w 32889572

[42] Lin YH Teng MMH. Comparing self-assessment, functional, and anthropometric techniques in predicting osteoporosis. Arch Osteoporos. (2020) 15. doi: 10.1007/s11657-020-00806-4 32812073

[43] Sakuma K Yamaguchi A. Sarcopenia and age-related endocrine function. Int J Endocrinol. (2012) 2012. doi: 10.1155/2012/127362 PMC3368374 22690213

[44] Cruz-Jentoft AJ Landi F Schneider SM Zúñiga C Arai H Boirie Y. Prevalence of and interventions for sarcopenia in ageing adults: A systematic review. Report of the International Sarcopenia Initiative (EWGSOP and IWGS). Age Ageing. (2014) 43:48–759. doi: 10.1093/ageing/afu115 PMC4204661 25241753

[45] Yusuf AA Hu Y Chandler D Crittenden DB Barron RL. Predictors of imminent risk of fracture in Medicare-enrolled men and women. Arch Osteoporos. (2020) 15:1–9. doi: 10.1007/s11657-020-00784-7 PMC7399683 32748034

[46] Bonafede M Shi N Barron R Li X Crittenden DB Chandler D. Predicting imminent risk for fracture in patients aged 50 or older with osteoporosis using US claims data. Arch Osteoporos. (2016) 11. doi: 10.1007/s11657-016-0280-5 PMC4967418 27475642

[47] Adachi JD Berger C Barron R Weycker D Anastassiades TP Davison KS. Predictors of imminent non-vertebral fracture in elderly women with osteoporosis, low bone mass, or a history of fracture, based on data from the population-based Canadian Multicentre Osteoporosis Study (CaMos). Arch Osteoporos. (2019) 14. doi: 10.1007/s11657-019-0598-x 31098708

[48] Roux C Briot K. Imminent fracture risk. Osteoporos Int. (2017) 28:1765–9. doi: 10.1007/s00198-017-3976-5 28236126

[49] Johansson H Siggeirsdóttir K Harvey NC Odén A Gudnason V Mccloskey E. Imminent risk of fracture after fracture Europe PMC Funders Group. Osteoporos Int. (2017) 28:775–80. doi: 10.1007/s00198-016-3868-0 PMC5338733 28028554

[50] Center JR Bliuc D Nguyen TV Eisman JA. Risk of subsequent fracture after low-trauma fracture in men and women. Jama. (2007) 297:387–94. doi: 10.1001/jama.297.4.387 17244835

[51] Hannan MT Weycker D McLean RR Sahni S Bornheimer R Barron R. Predictors of imminent risk of nonvertebral fracture in older, high-risk women: the framingham osteoporosis study. JBMR Plus. (2019) 3:1–10. doi: 10.1002/jbm4.10129 PMC6636767 31346561

[52] Camacho PM Petak SM Binkley N Diab DL Eldeiry LS Farooki A. American association of clinical endocrinologists/American college of endocrinology clinical practice guidelines for the diagnosis and treatment of postmenopausal osteoporosis-2020 update. Endocr Pract. (2020) 26:1–46. doi: 10.4158/GL-2020-0524SUPPL 32427503

